# RM-ADR: Resource Management Adaptive Data Rate for Mobile Application in LoRaWAN

**DOI:** 10.3390/s21237980

**Published:** 2021-11-30

**Authors:** Khola Anwar, Taj Rahman, Asim Zeb, Inayat Khan, Mahdi Zareei, Cesar Vargas-Rosales

**Affiliations:** 1 Department of Physical & Numerical Science, Qurtuba University of Science & Information Technology, Peshawar 25000, Pakistan; khola.anwer.shah@gmail.com; 2 Department of Computer Science, Abbottabad University of Science and Technology, Abbottabad 22500, Pakistan; asimzeb1@gmail.com; 3 Department of Computer Science, University of Buner, Buner 19290, Pakistan; inayat_khan@uop.edu.pk; 4 Tecnologico de Monterrey, Escuela de Ingenieria y Ciencias, Monterry 64849, Mexico; m.zareei@ieee.org (M.Z.); cvargas@tec.mx (C.V.-R.)

**Keywords:** LoRaWAN, internet of things, resource management, mobility, energy consumption, convergence time

## Abstract

LoRaWAN is renowned and a mostly supported technology for the Internet of Things, using an energy-efficient Adaptive Data Rate (ADR) to allocate resources (e.g., Spreading Factor (SF)) and Transmit Power (TP) to a large number of End Devices (EDs). When these EDs are mobile, the fixed SF allocation is not efficient owing to the sudden changes caused in the link conditions between the ED and the gateway. As a result of this situation, significant packet loss occurs, increasing the retransmissions from EDs. Therefore, we propose a Resource Management ADR (RM-ADR) at both ED and Network Sides (NS) by considering the packet transmission information and received power to address this issue. Through simulation results, RM-ADR showed improved performance compared to the state-of-the-art ADR techniques. The findings indicate a faster convergence time by minimizing packet loss ratio and retransmission in a mobile LoRaWAN network environment.

## 1. Introduction

**Figure 1:**
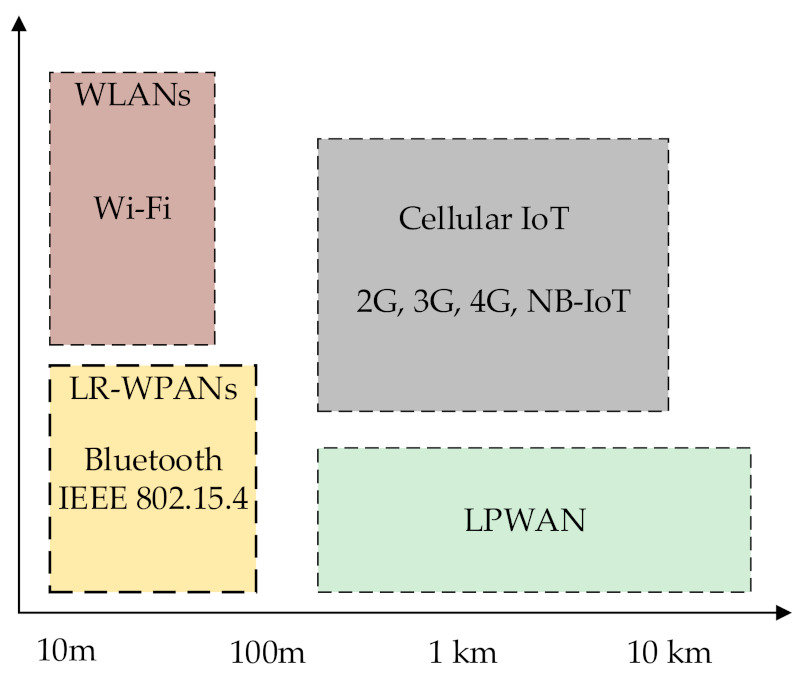
Internet of Things technologies.

Internet of Things (IoT) technologies are mainly categorized into Low-Rate Wireless Personal Area Networks (LRWPAN), cellular IoT, and Low-Power Wide-Area Networks (LPWANs), as shown in Figure 1. LPWAN technologies, such as Long-Term Evolution for Machines (LTE-M), SigFox, Long-Range Wide-Area Network (LoRaWAN), and Narrow Band (NB)-IoT, have emerged as licensed and unlicensed in the market. Among LPWAN technologies, LoRaWAN is the most widely used for IoT due to long-range communication and low-cost solutions [1,2,3]. Therefore, it has been widely adopted for IoT applications, offering long-range and ultra-low energy consumption with cheap solutions [4].

**Figure 2:**
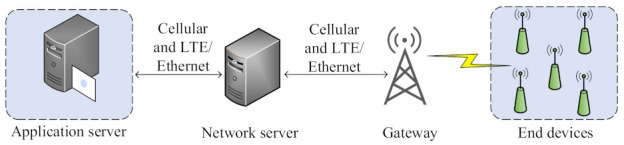
LoRa network architecture.

**Figure 3:**
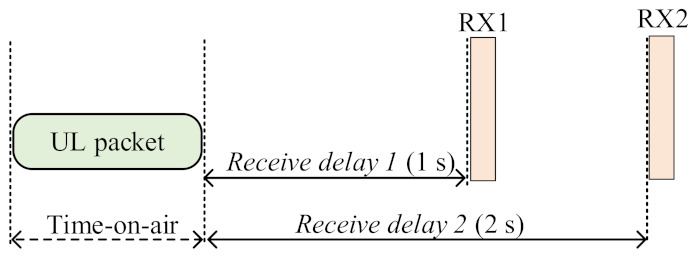
Receive windows operation in the confirmed mode of LoRaWAN.

Long-range (LoRa) describes the physical (PHY) layer characteristics (designed by the LoRa Alliance), while LoRaWAN is the Medium Access Control (MAC) layer. The basic LoRaWAN structure comprises end devices (EDs), a gateway (GW), a network server (NS), and an application server, as shown in Figure 2. The EDs involved in the communication always transmits an uplink (UL) to GW using the ALOHA mechanism by using spreading factors [SFs: 7 to 12]. Communication between ED and a GW can either be confirmed or unconfirmed. Confirmed mode is considered to be reliable, where ED anticipate a downlink (DL) acknowledgement (ACK) from NS after each uplink (UL), as shown in Figure 3. To receive ACK, ED opens a receive window 1 (RX1) after receive delay 1 (which is one second long) with the same SF and channel being used for UL packet. If the ED did not receive the ACK, it opens a second receive window (RX2) after receive delay 2 (which is two seconds long) with SF12 and a dedicated channel 869.525 MHz in EU-868 MHz frequency region. In the absence of ACK in both receive windows, ED retransmits the packet after a random time $RETRANSMIT_{TIMEOUT}$ (1–3 s). In contrast to confirmed mode, no ACK is required in the unconfirmed mode.

For resource allocation [e.g., SF and Transmit Power (TP)] to EDs, LoRaWAN adopts an adaptive data rate (ADR) [5,6]. However, it fails to adapt itself when the underlying environment is mobile, resulting in massive packet loss. Thus, it is recommended for static applications, such as metering [7]. Therefore, this proposes a resource management ADR (RM-ADR) at both ED- and NS-sides by considering packet transmission information and received power to alleviate this considerable packet loss by reducing the retransmission. The contributions of the proposed work are summarized below.

1. The proposed RM-ADR collects the packet transmission information and send it to the NS inside the LoRa frame header. Furthermore, the RM-ADR at the ED side allocates SF and TP based on the retransmission remaining, resulting in gaining connectivity with GW.
2. RM-ADR at the NS side make use of the packet transmission information and use the received power to assign both SF and TP to mobile end devices, resulting in a low packet loss arriving under the sensitivity thresholds at the GW.
3. RM-ADR at NS side greatly increases the efficiency of the Packet Delivery Ratio (PDR) by adapting itself to the varying conditions of the channels. Therefore, it enhances the convergence period when compared to state-of-the-art approaches.
4. Additionally, the proposed RM-ADR at NS side can help lower the energy consumption by reducing the retransmissions.

The paper is design as: Section 2 convey the literature review. Section 3 describes detailed working of the proposed resource management ADR (RM-ADR). Section 4 highlights the experimental study and analysis of the proposed RM-ADR, while the endmost Section 5 wind up the paper.

## 2. Related Work

**Figure 4:**
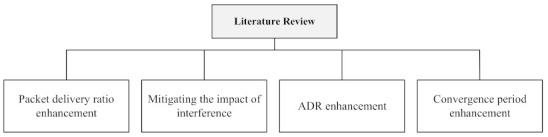
Categorization of the literature review.

Many existing studies have resolved LoRaWAN issues, such as PDR enhancement, mitigating the impact of interference, ADR enhancement, and convergence period enhancement. Therefore, we divide the existing literature into four categories, as shown in Figure 4. These categories are presented in the remaining of this section.

### 2.1. Packet Delivery Ratio (PDR) Enhancement

PDR can be enhanced by allocating the SFs using the sensitivities threshold related to GW [8,9]. The authors in [8] assigned a suitable SF to ED, concerning the received signal strength at the GW. Results showed enhanced PDR against the static SF assignment. However, the work in [8] was limited to only unconfirmed mode and completely ignored the confirmed mode of communication since it supports bidirectional communication and can influence the packet delivery ratio in the presence of ACK.

The work presented in [8,9] was enhanced [10] by analyzing both confirmed and unconfirmed communication modes. Apart from improving [8,9], authors in [10] also propose another approach for allocating SFs based on the ED sensitivity. Results of [10] compared with [8,9] showed improved performance in terms of PDR and lower end-to-end delay. Based on the simulation results presented in [10], it was recommended to use SF allocation to EDs based on the ED sensitivities due to the DL ACK message.

Another approach presented in [11] enhances the PDR using two methods: first, authors make groups of EDs based on their received signal strength indicators (RSSI) and assign a specific channel to each group. Secondly, the authors assign SFs to EDs in each group based on the RSSI. The proposed methods have decreased the collision by setting a suitable SF and a channel for a specific group.

The authors in [12] proposed two methods EXtending the performance of LoRa by suitable SF (EXploRa-SF) and EXtending the performance of LoRa by air-time (EXplora-AT) to enhances the PDR of LoRaWAN. EXploRa-SF was responsible for assigning SFs to the EDs in the network concerning the RSSI. In contrast, EXplora-AT tried to reduce the air-time by fairly allocating a lower SF based on an “ordered water-filling [13]” approach. When both the proposed methods were compared with the LoRaWAN-based typical ADR, the proposed method showed improved PDR results. This work [12] has been enhanced by [14] based on the K-means algorithm, which has increased the performance by 21% in terms of PDR.

### 2.2. Mitigating the Impact of Interference

An interference-aware SF assignment (I-ASF) [15] was proposed to eliminate intra-network interference. I-ASF initially allocated SF with GW sensitivity thresholds during the deployment phase. However, in the case of interference during the ongoing communication, I-ASF has allocated a satisfactory SF to reduce the interference impact, which has increased the PDR.

The authors studied the interference impact of a GW in the LoRaWAN network environment caused due to the UL packets transmission under multiple co-existing EDs [16]. The authors claimed that the aggregate interference that occurred to a GW does not follow a Gaussian distribution. The authors in [16] considered Class *A* EDs, where path loss, shadowing, and fading influence these EDs. Their results showed enhanced PDR by lowering the impact of interference.

Another work presented in [17] studied the impact of inter-technology interference and optimized the LoRaWAN network parameters. The authors proposed an analytical model to mitigate the external and internal interference between the LoRaWAN and IEEE 802.15.4 g devices under the Wi-sun system. Based on both technologies’ interference, the authors proposed two different optimization schemes to find the best possible SF and TP to meet the minimum reliability level [18,19]. Their methods showed improved performance in simulations. Therefore, they claimed that these methods could be used for planning interference-limited networks meeting the minimum required reliability.

The author in [20] evaluated the link quality LoRa-based wireless underground sensor networks (WUSNs) for the underground-to-aboveground (UG2AG) and aboveground-to-underground (AG2UG) communication. Using the in-situ data for the soil–air communications, the authors tested the channel model considering semiempirical path-loss models. The authors experimentally compared the impacts of the burial depths, the distances between EDs, and SF and TP parameters on the LoRa link quality. Their experimental results revealed that the channel is subtle to soil characteristics, underlying propagation environment, and SF and TP parameters. Furthermore, their experimental results revealed that received signal strength indicator (RSSI) and signal-to-noise ratio (SNR) are the best indicators for PDR.

The authors in [21] presented a feasibility study on the deployment of the LoRa for UWSNs. Their results revealed that the underwater-to-overwater (UW2OW) and overwater-to-underwater (OW2UW) channels are highly symmetric under different SF and TP parameters. Further their experimental results suggested that channel quality varies for the underwater-to-underwater (UW2UW) when the distance between the EDs is static.

The authors in [22] aimed to study the reliability of the LoRa and LoRaWAN of vehicles at a different speed. Their experimental results with a speed of 90 km/h revealed satisfactory performance. Therefore, the authors recommended using LoRaWAN for high-speed cars and trains with acceptable performance.

Ref. [23] studied the performance of the transmission parameters (i.e., SF and TP) for sailing monitoring systems. The experiments were conducted for different transmission parameters and distances. Based on the study, optimal SF and TP parameters were identified that can satisfy the application requirements.

In [24], the authors evaluated the performance of Smart Cities by using Vehicle-to-Cloud interface with OBD-II (On-Board Diagnostic) communication, 3G/4G connectivity, and LoRaWAN. First, the authors presented constraints regarding smart city use and further evaluated the probability that an ED can transmit data to their proposed architecture. Their experimental results revealed the feasibility of their proposed infrastructure for the smart city scenario.

The authors in [25] presented a CAN-BUS prototype considering a vehicle to grid (V2G) communication based on intra-vehicle data collection and interchange to aggregators. The data were gathered using the CAN-BUS prototype, connected with EDs and GW by connecting web services in a cloud-based NS. In addition, the authors tested LoRaWAN using frequency EU-868 MHz. The analysis was carried out with the help of an in-house 3D Ray Launching (3D-RL) code to obtain optimal received power distribution, interference, and other time-domain parameters in a large and complex LoRaWAN environment. Their results show that the system is efficient for monitoring purposes at the bus stops or the complex infrastructure of the buildings.

### 2.3. ADR Enhancement

The authors in [26] proposes an ADR using the Standard Deviation of averaged SNR of *P* packets (i.e., P=20). Their method shows improved performance in terms of packet delivery ratio by effectively determining the SF and TP. However, the authors in [26] failed to improve the convergence period of the ADR.

An improved ADR (IADR) was proposed in [27] to address the issues related to the initial SF 12 assignment during the deployment phase of the typical ADR. IADR assigned all SFs to EDs involved in the communication at the time when the network deployment based on the received signal strength. The performance results of the IADR showed enhanced PDR compared to ADR.

Recently, the authors proposed a retransmission-assisted ADR (RM-ADR) in [28] to improve the PDR by reducing the retransmission attempts of the ED in UL. Their results show improved performance in PDR, energy consumption, and convergence period compared to the existing state-of-the-art ADRs mechanisms.

### 2.4. Convergence Period Enhancement

The authors in [29] proposed ADR at ED- and NS side. Their ADR at the ED side is simple, which works by simply taking the ratio of the uplink and downlink of last 5 packets. Their ADR at the NS side is responsible to manage both SF and TP after the five packets. Their simulation outcomes reveal improved performance. However, their study considered a static environment.

Furthermore, the authors in [30] proposed two ADRs: Gaussian-ADR (G-ADR) and Exponential moving average-ADR (EMA-ADR). The primary purpose of their proposed methods was to reduce the convergence time and improve the PDR of the LoRaWAN network. Both their proposed ADRs significantly improved the PDR, convergence time, and energy consumption.

## 3. Working of the Proposed RM-ADR

The proposed RM-ADR manages the resources (i.e., SF and TP) at ED- and NS-sides. In the rest of this section, RM-ADR is presented.

### 3.1. RM-ADR at ED Side

**Figure 5:**
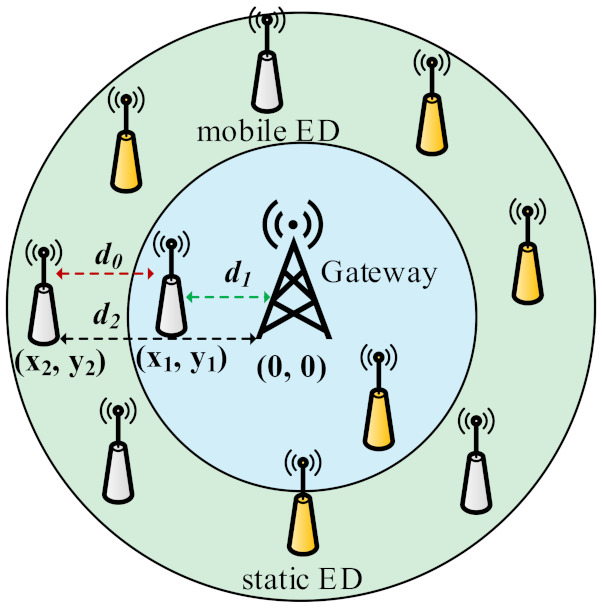
Distance between current and previous positions of the end device.

To determine, an ED is mobile or static, we have used a method shown in [3]. The method described in [3] computes the distance ($d_{o}$) between the current ($d_{2}$) and previous ($d_{1}$) positions of the ED to determine the mobility using Euclidean distance method, as shown in Figure 5 [3]. In Figure 5, two types of ED are shown: static and mobile. The previous distance (i.e., $d_{1}$) between the GW and ED is represented with green arrow, while the current position ($d_{2}$) is represented with black arrow. When both $d_{1}$ and $d_{2}$ are determined, $d_{o}$ (represented with red arrow) is computed by taking difference of both. The resultant value can be negative or positive, showing that the ED has moved near to or far from GW. Finally, the $d_{o}$ is compared to a threshold (i.e., α = 20 m [3]). If the condition holds, the ED is determined as mobile. For a mobile ED, several UL transmissions are counted using a counter (Tx_CNT) when $Fcnt_{cur}$ (a sequence number associated with UL packet) equals to $Fcnt_{prev}$. In this work, we have modified the LoRa frame header to accommodate Tx_CNT information, which contains 1 byte.

In contrast to mobile ED, the proposed RM-ADR assigns SF and TP at the ED side when several retransmissions (Rtx_CNT) are equal to γ (it is a threshold, which is set to 2 [10,31]) . The choice of γ = 2 is chosen to prevent further retransmission from this ED and reduce the interference. Then, the ED (either mobile or static) obtains a random channel and transmits an uplink to GW (see Algorithm 1).
**Algorithm 1:** The proposed RM-ADR at the ED side.
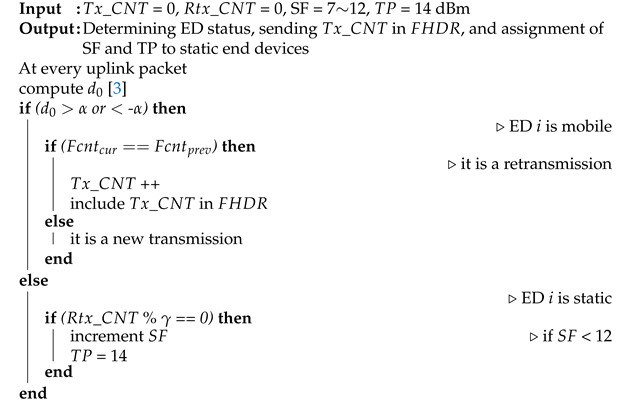


### 3.2. RM-ADR at NS Side

Resource Management Adaptive Data Rate (RM-ADR) at the NS side is responsible for allocating resources to mobile EDs. At the start of the simulation, each ED is assigned an SF12, as suggested by the typical ADR method in [5]. Then, each ED enables ADRACKReq (MAC command, which allows the DL ACK from the NS in the confirmed mode of communication) in the FHDR and transmits a packet to GW containing Tx_CNT. The proposed RM-ADR at the NS side is initiated if ADRACKReq is being enabled, as shown in Algorithm 2.

The proposed RM-ADR extracted the Tx_CNT from the frame header (FHDR) and compared it to a packet transmission threshold ($Tx_{threshold}$ = 3). The choice of $Tx_{threshold}$ = 3 is to reduce the retransmission of packets from ED. When this condition is true, the proposed RM-ADR computes new communication parameters based on the GW sensitivity contained in Table 1.

Finally, the proposed ADR assigns the ED the lowest possible SF above the GW sensitivity. The advantage of this procedure is to reduce the chances of collisions by lowering the ToA.

As an example of this SF assignment method, consider the following scenario: a $P_{rx}$ of −137 dBm received at the GW. In this scenario, based on the $GW_{sen}$ sensitivity values contained in Table 1, SF9 can be considered too low; however, SF ∈ [10, 11, 12] would allow us to receive packets from EDs. We configured the EDs to use SF10 since we are interested in decreasing the ToA in general.

In contrast, the typical ADR [5] is initiated if the ED is static, as shown in Algorithm 2.
**Algorithm 2:** Resource management for mobile end devices.
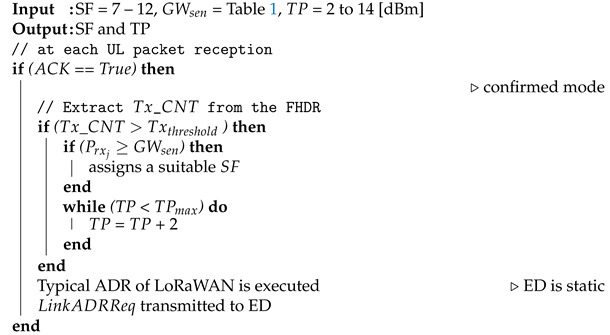


### 3.3. Integration of the RM-ADR LoRaWAN

**Figure 6:**
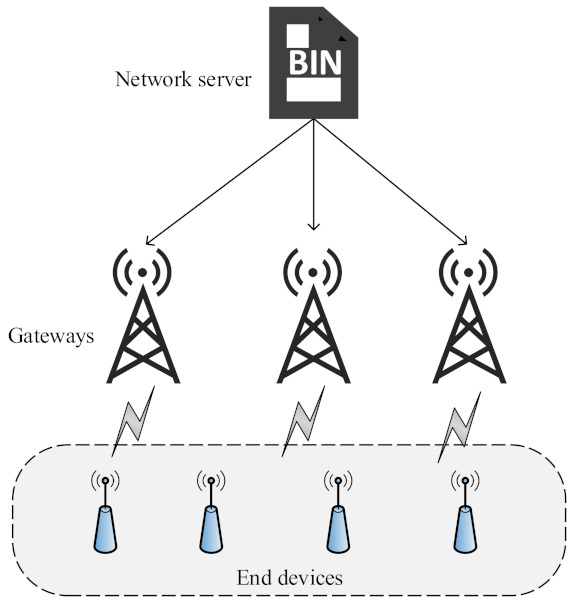
Network server process of firmware update in LoRaWAN.

The proposed RM-ADR at the ED side can easily be integrated at the ED using firmware update over the air process, as shown in Figure 6. In contrast, the NS side RM-ADR can be integrated in the NS as a routine maintenance.

## 4. Experimental Analysis of the Proposed RM-ADR

In the current section , we evaluate the proposed RM-ADR compared to ADR [5] and Gaussian-ADR (G-ADR) [30]. The performance study comprises packet delivery ratio (PDR), packet loss ratio (PLR), convergence period, and energy consumption. The performance is evaluated using network simulator (ns)-3 [34].

In addition, this work considers both intra- and inter-SF interferences, similar to [8,10,15]. Based on Equation (1), when a packet is at the GW with $SF_{\left(i,j\right)}$, it can be successfully received if the interference power is larger than the threshold $\beta_{\left(i,j\right)}$. In contrast, a packet can be considered lost.

(1) $$\beta_{\left(i,j\right)}=\left[\begin{array}{cccccc} 6 & -16 & -18 & -19 & -19 & -19\\ -24 & 6 & -20 & -22 & -22 & -22\\ -27 & -27 & 6 & -23 & -25 & -25\\ -30 & -30 & -30 & 6 & -26 & -28\\ -33 & -33 & -33 & -33 & 6 & -29\\ -36 & -36 & -36 & -36 & -36 & 6 \end{array}\right]$$

### 4.1. Simulation Environment

In this work, we consider Class *A* end devices due to their applicability in the pet-tracking application and energy efficiency [35]. These EDs are distributed randomly within 5-km range in a single GW environment. The EDs comply with log-distance and shadowing models along with the random-walk mobility model. The simulation parameters used in the paper, are mentioned in Table 2.

Furthermore, a pet-tracking application with diverse requirements is considered in this work, as highlighted in Table 3 [7,28]. The packet size contains 9 bytes of PHY/MAC header size.

### 4.2. Performance Analysis

#### 4.2.1. Over All Network Performance

**Figure 7:**
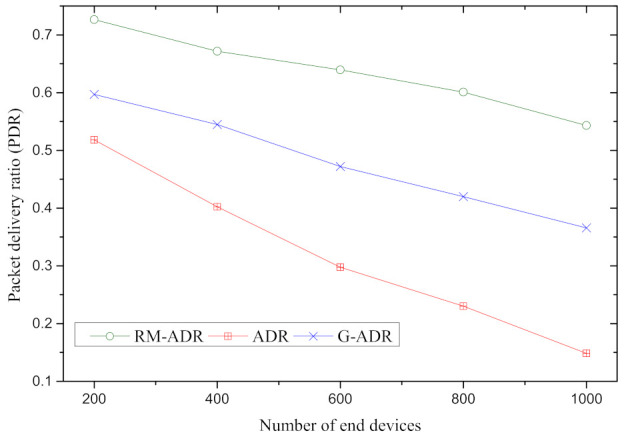
PDR of the proposed RM-ADR and existing ADRs.

PDR of the proposed RM-ADR, ADR, and G-ADR is highlighted in Figure 7 for a different number of mobile end devices. In Figure 7, the PDR decreases when the end devices are increased owing to the increase in both UL and DL. As a result, interference occurs, and packets are lost. These lost packets are retransmitted with old SF and TP configuration, resulting in packets arriving under the sensitivity in ADR and G-ADR. However, G-ADR performance is much better than ADR because it configures the SF and TP after 5 UL packets are received at the NS. In contrast to ADR and G-ADR, the proposed RM-ADR performance is better owing to the SF and TP adjustment based on the number of transmissions performed by the ED. This method reduces the chances of retransmission, resulting in improved PDR.

**Figure 8:**
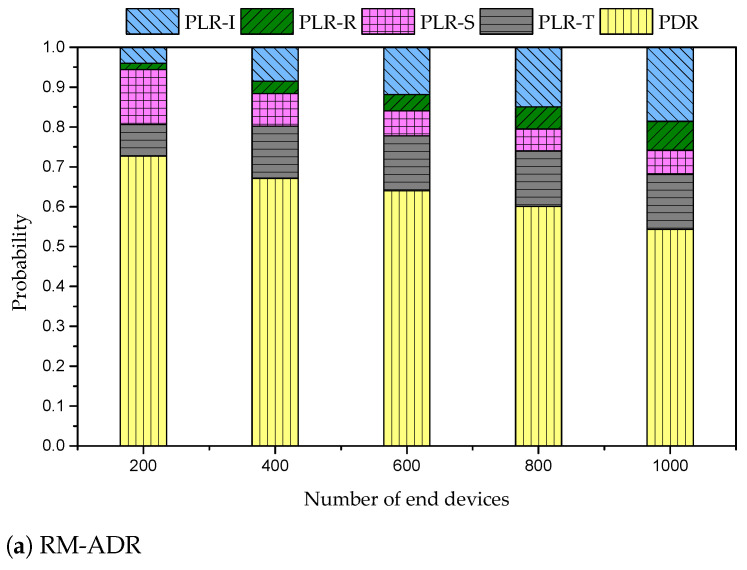

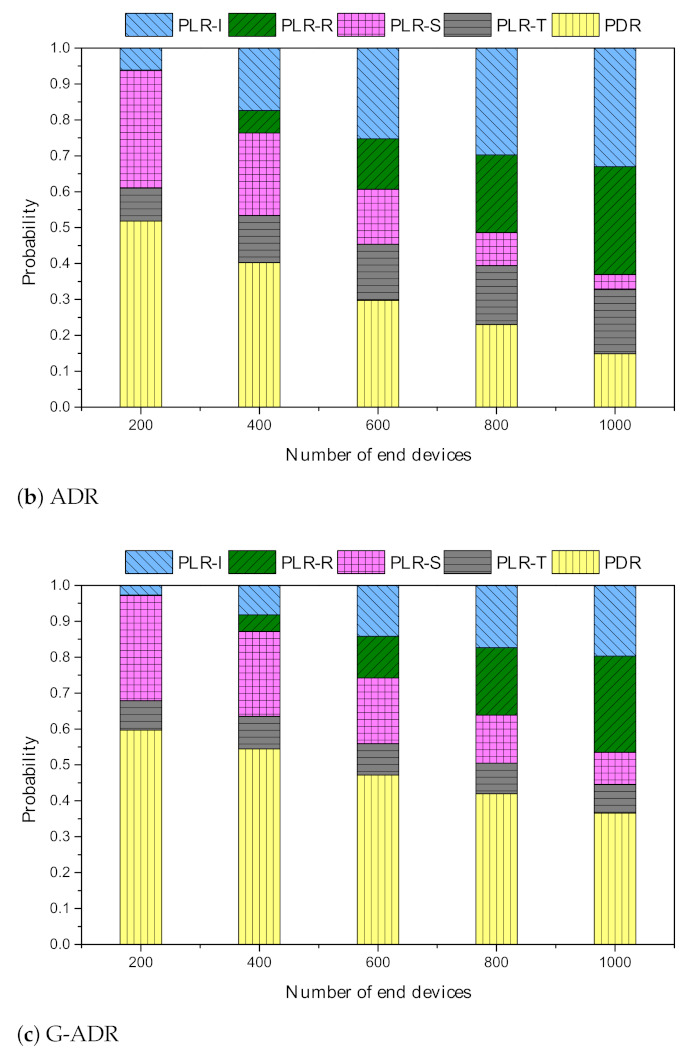
Probability of packet delivery and loss ratios (PDR and PLRs) of the proposed RM-ADR, ADR, and G-ADR.

Figure 8 shows the probability of ratios of PDR and packet loss ratio (PLR) of the proposed RM-ADR, ADR, and G-ADR. For example, from Figure 8 it is clear that the probability of both PDR and PLR is 1.

In Figure 8, PLR-I represents the PLR caused due to the intra- and inter-SF interferences. The impact of PLR-I is significant in ADR owing to the retransmission of packets with high SF (e.g., SF 10, 11, and 12). In contrast, the PLR-I of RM-ADR is recorded similar to that of G-ADR.

In Figure 8, PLR-R shows the PLR caused owing to reception paths being busy at the GW while demodulating the incoming packets. This work only uses the default three channels for UL (i.e., 868.1, 868.3, and 868.5 MHz) at the GW with 8 parallel reception paths to demodulate 8 different packets with either SF [28]. However, when these paths are busy, an incoming packet cannot be demodulated, resulting in PLR. The PLR-R impact is high in ADR and G-ADR due to a high number of retransmissions. Since the lost packets are retransmitted, which increase the uplink traffic. In contrast, the PLR-R impact for the proposed RM-ADR is low due to SF and TP adjustment based on the ED packet transmission information.

PLR-S in Figure 8 is defined as when the packets are lost at the GW owing to arriving under the required sensitivity threshold (these thresholds of all SFs are defined in Table 1). PLR-S is decreasing in both ADR and G-ADR, while it is almost constant in the proposed RM-ADR.

Finally, PLR-T in Figure 8 occurs when an ongoing packet is lost due to the ACK transmission from the GW. By default, the GW implements ACK priority mechanism at the GW. This impact is almost similar in all ADRs. For more in-depth analysis of these PLRs in a confirmed mode, refer to the papers in [28,30].

#### 4.2.2. Average Energy Consumption

**Figure 9:**
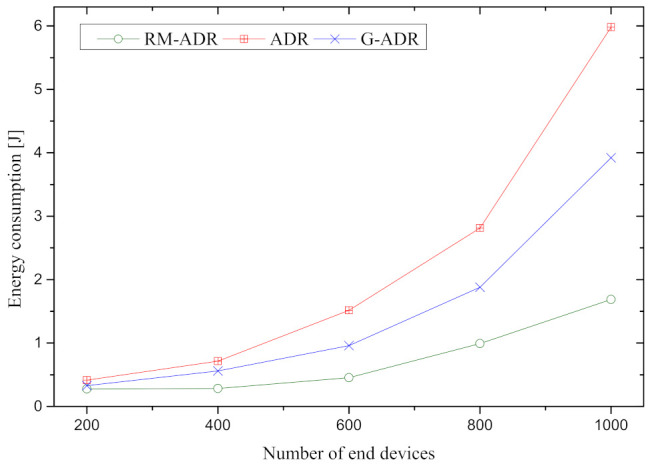
Energy Consumption in Joules [J] of the proposed RM-ADR and existing ADRs.

Figure 9 depicts the average energy consumption of the proposed RM-ADR and state-of-the-art ADR schemes. In general, when the number of EDs increase, the energy consumption for all ADRs is increased. This is because, during the network deployment, all ADRs use SF = 12, which produces substantial interference owing to the high ToA.

However, energy consumption is significant in both ADR and G-ADR. Furthermore, the ADR’s energy usage is greater relative to the G-ADR and RM-ADR due to the number of retransmissions. For example, if a packet is lost during communication, it is retransmitted 7 times with same SF and TP. In the case of ADR, this retransmission consumes a substantial amount of energy. As a result, we believe that the high SF, TP, ToA, and retransmission in ADR and G-ADR is high in energy consumption.

In contrast, the proposed RM-ADR energy consumption is observed lower when compared to both ADR and G-ADR because it assigns an appropriate SF and TP. Therefore, energy consumption is lower in the case of the proposed RM-ADR compared to ADR and G-ADR.

#### 4.2.3. Convergence Period

**Figure 10:**
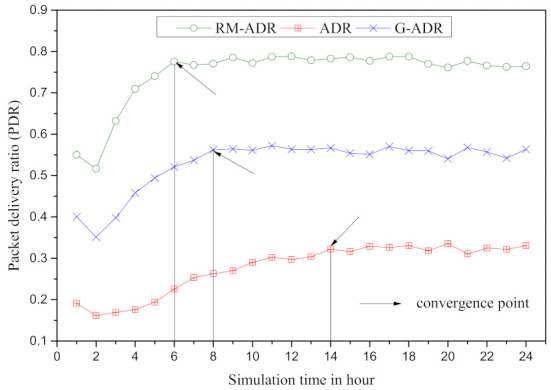
Convergence Period of the proposed RM-ADR and existing ADRs.

The convergence period for proposed RM-ADR, ADR, and G-ADR is presented for N=500 in Figure 10. In Figure 10, RM-ADR, G-ADR, and ADR suffer from convergence periods of 6, 8, and 14 h.

The ADR suffers from a long convergence time in Figure 10 because it is unable to adjust to the fluctuating channel condition produced by the ED movement [37].

On the other hand, the convergence of RM-ADR is 2, and 8 h lower than G-ADR and ADR, respectively. The proposed RM-ADR adjusts both SF and TP based on the transmission information shared by the ED with NS, which accounts for the short convergence time.

#### 4.2.4. Final Sf Use by EDs

**Figure 11:**
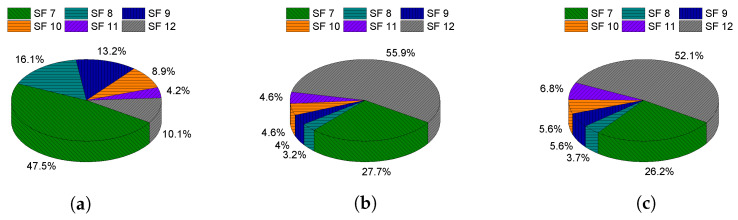
End devices using final spreading factors (in percentage) with N=500 in the case of mobile EDs. (**a**) Proposed RM-ADR; (**b**) ADR; (**c**) G-ADR.

Figure 11 and Table 4 show the final SF use by the EDs with N=500 for ADR, G-ADR, and the proposed RM-ADR. It can be seen that ADR and G-ADR use SF 12, the most used SF among the other SFs, resulting in high time-on-air. This high time-on-air causes significant intra- and inter-SF interferences. As a result, it is leading to high packet loss. In contrast, only 10.1% of the total N=500 EDs use SF 12 in the case of the proposed RM-ADR. Therefore, the proposed RM-ADR uses the packet transmission information and received power and accurately determines better SF and TP compared to the existing ADR approaches.

## 5. Conclusions

In this study, we proposed ED- and NS side ADRs for spreading factor and transmit power management. RM-ADR implemented at the ED side counts the number of transmissions from each ED and sent to NS contained in LoRa frame header and assigned SF and TP to the ED based on retransmission information. On the other hand, the proposed RM-ADR at the NS side extracted the number of transmission information from the LoRa frame header, compared it to a packet transmission threshold, and assigned SF and TP parameters on the received power. Compared to state-of-the-art ADRs schemes, the proposed RM-ADR showed improved results in convergence time, PDR and energy consumption.

## Figures and Tables

**Table 1:** ED and GW sensitivities threshold [30,32,33].

Sensitivity Types	SF7	SF8	SF9	SF10	SF11	SF12
**ED ($ED_{sen}$) [dBm]**	−124.0	−127.0	−130.0	−133.0	−135.0	−137.0
**GW ($GW_{sen}$) [dBm]**	−130.0	−132.5	−135.0	−137.5	−140.0	−142.5

**Table 2:** Parameters used in the simulation study.

Parameter	Value
Simulation time [h]	24
Number of transmission	8
Path-loss exponent	3.76 [36]
Loss model	log-distance
Mobility model	random walk 2-D [3,30]
ED movement speed [m/s]	0.5∼1.5 [3,30]
Transmit power [dBm]	2∼14
Frequency region	EU-868

**Table 3:** Pet-tracking application with various requirements [7,28].

Considered Application	Proposed by	Packet Interval/Day	Payload Size [Bytes]
Pet-tracking	Semtech [7]	144 (for a single ED)	30

**Table 4:** End devices using final spreading factors (in percentage) with N=500 in the case of mobile EDs.

Scheme	SF7	SF8	SF9	SF10	SF11	SF12
ADR	33.7	2.3	2.9	2.9	2.4	55.8
G-ADR	26.2	3.7	5.6	5.6	6.8	52.1
Proposed RM-ADR	47.5	16.1	13.2	8.9	4.2	10.1

## Data Availability

Not applicable.

## References

[1] Gomez C., Veras J.C., Vidal R., Casals L., Paradells J. (2019). A sigfox energy consumption model. Sensors.

[2] Chalapati S. (2018). Comparison Of LPWA Technologies And Realizable Use Cases. https://www.nctatechnicalpapers.com/Paper/2018/2018-comparison-of-lpwa-technologies-and-realizable-use-cases.

[3] Farhad A., Kim D.H., Kim B.H., Mohammed A.F.Y., Pyun J.Y. (2020). Mobility-Aware Resource Assignment to IoT Applications in Long-Range Wide Area Networks. IEEE Access.

[4] Siddiqi T.R., Ning H., Ping H., Mahmood Z. (2016). DPCA: Data prioritization and capacity assignment in wireless sensor networks. IEEE Access.

[5] ETSI (2018). System Reference Document (SRdoc); Technical Characteristics for Low Power Wide Area Networks and Chirp Spread Spectrum (LPWAN-CSS) Operating in the UHF Spectrum below 1 GHz; ETSI TR 103 526 V1.1.1 (2018-04). https://www.etsi.org/deliver/etsi_tr/103500_103599/103526/01.01.01_60/tr_103526v010101p.pdf.

[6] Li S., Raza U., Khan A. How Agile is the Adaptive Data Rate Mechanism of LoRaWAN. Proceedings of the IEEE Global Communications Conference (GLOBECOM).

[7] SEMTECH LoRaWAN® Mobile Applications: Blind ADR. https://lora-developers.semtech.com/uploads/documents/files/LoRaWAN_Mobile_Apps-Blind_ADR_Downloadable.pdf.

[8] Magrin D., Centenaro M., Vangelista L. Performance evaluation of LoRa networks in a smart city scenario. Proceedings of the IEEE International Conference on communications (ICC).

[9] Magrin D., Capuzzo M., Zanella A. (2019). A Thorough Study of LoRaWAN Performance Under Different Parameter Settings. IEEE Internet Things J..

[10] Farhad A., Kim D.H., Pyun J.Y. (2020). Resource allocation to massive internet of things in lorawans. Sensors.

[11] Reynders B., Wang Q., Tuset-Peiro P., Vilajosana X., Pollin S. (2018). Improving reliability and scalability of lorawans through lightweight scheduling. IEEE Internet Things J..

[12] Cuomo F., Campo M., Caponi A., Bianchi G., Rossini G., Pisani P. EXPLoRa: Extending the performance of LoRa by suitable spreading factor allocations. Proceedings of the 2017 IEEE 13th International Conference on Wireless and Mobile Computing, Networking and Communications (WiMob).

[13] Bianchi G., Cuomo F., Garlisi D., Tinnirello I. (2019). Capture aware sequential waterfilling for LoraWAN adaptive data rate. arXiv.

[14] Cuomo F., Gámez J.C.C., Maurizio A., Scipione L., Campo M., Caponi A., Bianchi G., Rossini G., Pisani P. Towards traffic-oriented spreading factor allocations in LoRaWAN systems. Proceedings of the 2018 17th Annual Mediterranean Ad Hoc Networking Workshop (Med-Hoc-Net).

[15] Farhad A., Kim D., Sthapit P., Pyun J. Interference-Aware Spreading Factor Assignment Scheme for the Massive LoRaWAN Network. Proceedings of the 2019 International Conference on Electronics, Information, and Communication (ICEIC).

[16] Irio L., Oliveira R. Modeling the Interference caused to a LoRaWAN Gateway due to Uplink Transmissions. Proceedings of the 2019 Eleventh International Conference on Ubiquitous and Future Networks (ICUFN).

[17] Hoeller A., Souza R.D., Alves H., López O.L.A., Montejo-Sánchez S., Pellenz M.E. (2019). Optimum LoRaWAN configuration under Wi-SUN interference. IEEE Access.

[18] Aggarwal S., Nasipuri A. Improving Scalability of LoRaWAN Networks by Spreading Factor Distribution. Proceedings of the SoutheastCon 2021.

[19] Coutaud U., Heusse M., Tourancheau B. (2021). LoRa Channel Characterization for Flexible and High Reliability Adaptive Data Rate in Multiple Gateways Networks. Computers.

[20] Lin K., Hao T. (2020). Experimental Link Quality Analysis for LoRa-based Wireless Underground Sensor Networks. IEEE Internet Things J..

[21] Lin K., Hao T., Zheng W., He W. Analysis of LoRa Link Quality for Underwater Wireless Sensor Networks: A Semi-empirical Study. Proceedings of the 2019 IEEE Asia-Pacific Microwave Conference (APMC).

[22] Di Renzone G., Parrino S., Peruzzi G., Pozzebon A. LoRaWAN in Motion: Preliminary Tests for Real Time Low Power Data Gathering from Vehicles. Proceedings of the 2021 IEEE International Workshop on Metrology for Automotive (MetroAutomotive).

[23] Li L., Ren J., Zhu Q. On the application of LoRa LPWAN technology in Sailing Monitoring System. Proceedings of the 2017 13th Annual Conference on Wireless On-demand Network Systems and Services (WONS).

[24] Ferrari P., Sisinni E., Carvalho D.F., Depari A., Signoretti G., Silva M., Silva I., Silva D. On the use of LoRaWAN for the Internet of Intelligent Vehicles in Smart City scenarios. Proceedings of the 2020 IEEE Sensors Applications Symposium (SAS).

[25] Klaina H., Guembe I.P., Lopez-Iturri P., Astrain J.J., Azpilicueta L., Aghzout O., Alejos A.V., Falcone F. (2020). Aggregator to electric vehicle LoRaWAN based communication analysis in vehicle-to-grid systems in smart cities. IEEE Access.

[26] Peruzzo A., Vangelista L. A power efficient adaptive data rate algorithm for LoRaWAN networks. Proceedings of the 2018 21st International Symposium on Wireless Personal Multimedia Communications (WPMC).

[27] Farhad A., Kim D.H., Kwon D., Pyun J.Y. An Improved Adaptive Data Rate for LoRaWAN Networks. Proceedings of the 2020 IEEE International Conference on Consumer Electronics-Asia (ICCE-Asia).

[28] Farhad A., Kim D.H., Pyun J.Y. (2021). R-ARM: Retransmission-Assisted Resource Management in LoRaWAN for the Internet of Things. IEEE Internet Things J..

[29] Finnegan J., Farrell R., Brown S. (2020). Analysis and Enhancement of the LoRaWAN Adaptive Data Rate Scheme. IEEE Internet Things J..

[30] Farhad A., Kim D.H., Subedi S., Pyun J.Y. (2020). Enhanced LoRaWAN Adaptive Data Rate for Mobile Internet of Things Devices. Sensors.

[31] Feltrin L., Buratti C., Vinciarelli E., De Bonis R., Verdone R. (2018). LoRaWAN: Evaluation of link-and system-level performance. IEEE Internet Things J..

[32] Semtech (2017). Semtech SX1301 WIRELESS & SENSING PRODUCTS Datasheet. https://www.semtech.com/products/wireless-rf/lora-gateways/sx1301.

[33] Semtech (2017). Semtech WIRELESS & SENSING PRODUCTS, Sx1272. https://www.semtech.com/products/wireless-rf/lora-transceivers/sx1272.

[34] Network Simulator (ns)-3. https://www.nsnam.org/.

[35] Farhad A., Kim D.H., Yoon J.S., Pyun J.Y. Feasibility Study of the LoRaWAN blind Adaptive Data Rate. Proceedings of the 2021 Twelfth International Conference on Ubiquitous and Future Networks (ICUFN).

[36] Farhad A., Kim D., Pyun J. Scalability of LoRaWAN in an Urban Environment: A Simulation Study. Proceedings of the Eleventh International Conference on Ubiquitous and Future Networks (ICUFN).

[37] Anwar K., Rahman T., Zeb A., Saeed Y., Khan M.A., Khan I., Ahmad S., Abdelgawad A.E., Abdollahian M. (2021). Improving the Convergence Period of Adaptive Data Rate in a Long Range Wide Area Network for the Internet of Things Devices. Energies.

